# Inhibition of STAT3/PD-L1 and Activation of miR193a-5p Are Critically Involved in Apoptotic Effect of Compound K in Prostate Cancer Cells

**DOI:** 10.3390/cells10082151

**Published:** 2021-08-20

**Authors:** Jae-Hee Lee, Dae-Young Lee, Hyo-Jung Lee, Eunji Im, Deok-Yong Sim, Ji-Eon Park, Woon-Yi Park, Bum-Sang Shim, Sung-Hoon Kim

**Affiliations:** 1 Department of Korean Pathology, College of Korean Medicine, Kyung Hee University, Seoul 02447, Korea; navbou0@naver.com (J.-H.L.); hyonice77@naver.com (H.-J.L.); ji4137@naver.com (E.I.); simdy0821@naver.com (D.-Y.S.); wdnk77@naver.com (J.-E.P.); wy1319@naver.com (W.-Y.P.); 2 Department of Herbal Crop Research, National Institute of Horticultural and Herbal Science, Rural Development Administration (RDA), Eumseong 27709, Korea; dylee0809@gmail.com

**Keywords:** prostate cancer, compound K, apoptosis, STAT3, PD-L1

## Abstract

Since the signal transducer and activator of transcription 3 (STAT3)/programmed death-ligand 1 (PD-L1) signaling plays an important role in tumor-immune microenvironments, in the present study, the role of STAT3/PD-L1 signaling in the apoptotic mechanism of an active ginseng saponin metabolite compound K (CK) was investigated in human prostate cancer cells. Here, CK exerted significant cytotoxicity without hurting RWPE1 normal prostate epithelial cells, increased sub-G1 and cleavage of Poly ADP-ribose polymerase (PARP) and attenuated the expression of pro-PARP and Pro-cysteine aspartyl-specific protease3 (pro-caspase-3) in LANCap, PC-3 and DU145 cells. Further, CK attenuated the expression of p-STAT3 and PD-L1 in DU145 cells along with disrupted the binding of STAT3 to PD-L1. Furthermore, CK effectively abrogated the expression of p-STAT3 and PD-L1 in interferon-gamma (INF-γ)-stimulated DU145cells. Additionally, CK suppressed the expression of vascular endothelial growth factor (VEGF), transforming growth factor-β (TGF-β), interleukin 6 (IL-6) and interleukin 10 (IL-10) as immune escape-related genes in DU145 cells. Likewise, as STAT3 targets genes, the expression of CyclinD1, c-Myc and B-cell lymphoma-extra-large (Bcl-xL) was attenuated in CK-treated DU145 cells. Notably, CK upregulated the expression of microRNA193a-5p (miR193a-5p) in DU145 cells. Consistently, miR193a-5p mimic suppressed p-STAT3, PD-L1 and pro-PARP, while miR193a-5p inhibitor reversed the ability of CK to attenuate the expression of p-STAT3, PD-L1 and pro-PARP in DU145 cells. Taken together, these findings support evidence that CK induces apoptosis via the activation of miR193a-5p and inhibition of PD-L1 and STAT3 signaling in prostate cancer cells.

## 1. Introduction

Programmed death-1 (PD-1) and its ligand PD-L1 are defined immune checkpoint molecules [1]. It is well documented that PD-L1 overexpressed in cancer cells binds to PD-1 in T cells and induces inactivation of T cells, leading to the immune escape in several cancers [2,3,4]. Accumulating evidence reveals that STAT3 is phosphorylated for tumor progression and inflammation, immunosuppression and immune escape [5,6]. Moreover, immune escape in tumor cells is closely associated with tumor progression [7,8]. Emerging evidence reveals that the STAT3 and PD-L1 are critically involved in cancer proliferation, cancer progression and immunosuppression [6,9,10]. Hence, a PD-L1 and/or STAT3 blockade with anti-PD-L1 or anti-STAT3 is considered as a potent treatment strategy in several cancers [11,12] since the overexpression of PD-L1 and STAT3 is observed in melanoma, pancreatic, lung and other types of cancer cells [13,14]. Nonetheless, it is not successful in prostate cancer therapy so far, though prostate cancer is known to be the most common type of cancer in men worldwide [15].

Recently some compounds are targeting PD-L1 and/or STAT3 for cancer therapy [16,17,18]. Manling et al. reported that ginsenoside RK1 significantly inhibited the proliferation and induced apoptosis by regulating the NF-κB pathway through controlling PD-L1 expression in lung adenoma cells [19], and Fan et al. suggested that niclosamide enhances the efficacy of PD-1/PD-L1 immune checkpoint blockades via the inhibition of STAT3 and PD-L1 signaling [20]. In addition, baicalein and baicalin were reported to increase antitumor immunity and inhibit tumor growth via the suppression of STAT3 and PD-L1 in hepatocellular carcinoma cells [21]. Furthermore, brusatol inhibited STAT3 mediated metastasis in liver cancer [22].

Similarly, compound K (CK; 20-*O*-*β*-D-glucopyranosyl-20(S)-protopanaxadiol) is a major metabolite of processed *panax ginseng* products, such as black ginseng [23], and is reported to exhibit anti-cancer [24] and anti-angiogenic effects in human umbilical vein endothelial cells [25]. Nevertheless, the underlying antitumor mechanism of CK has not been fully understood in prostate cancers so far. Thus, in the current study, the apoptotic mechanism of CK in association with STAT3 and PD-L1 signaling via the upregulation of miR193a-5p was elucidated in prostate cancer cells.

## 2. Materials and Methods

### 2.1. Compound K

**Figure 1:**
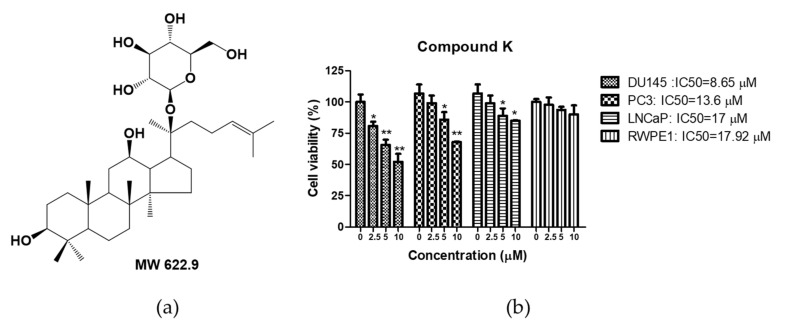
Effect of compound K on cytotoxicity in DU145, PC3 and LNCaP cells. (**a**) Chemical structure of compound K (CK). Molecular weight = 622.9 (**b**) DU145, PC3, LNCaP and RWPE1 cells were treated with various concentrations of CK (0, 2.5, 5 and 10 μM) for 24 h, and cell viability was evaluated by MTT assay. Data represent means ± SD. * *p* < 0.05, ** *p* < 0.01 versus untreated control.

Compound K (CK) was isolated and purified from black ginseng roots by a series of chromatography procedures by the Natural Product Chemistry and Metabolomics Laboratory in Rural Development Administration (RDA) (Eumseong, Korea), and the chemical structure was identified by comparing the spectroscopic data (UPLC-QTOF/MS) with the literature [23]. CK (Figure 1a), supplied by Dr. Dae Young Lee from the RDA, was diluted in DMSO for the next experiments and stored in a deep freezer.

### 2.2. Cell Culture

Human prostate cancer cells DU145, PC3, LNCaP and human normal prostate epithelial RWPE1 cells were bought from American Type Culture Collection (ATCC). DU145, PC3 and LNCaP cells were maintained in RPMI1640 with 10% FBS and 1% antibiotic (Welgene, Gyeongsan-si, Korea). RWPE-1 cells were cultivated using Keratinocyte Serum-Free Medium supplemented with 0.05 mg/mL bovine pituitary extract, 5 ng/mL recombinant human epidermal growth factor (EGF) and 1% antibiotic (Invitrogen, Grand Island, NY, USA).

### 2.3. Cytotoxicity Assay

The cytotoxicity of CK was assessed by 3-(4,5-dimethylthiazol-2-yl)-2,5-diphenyltetrazolium bromide (MTT) assay. Briefly, DU145, PC3, LNCaP and RWPE1 cells (1 × 10^4^ cells/well) were exposed to various concentrations of CK for 24 h, incubated with MTT (1 mg/mL) (Sigma Chemical, St. Louis, MO, USA) for 2 h and then treated with MTT lysis solution overnight. The optical density (OD) was determined by using a microplate reader (Molecular Devices Co., San Jose, CA, USA) at 570 nm for the evaluation of cell viability as a percentage of viable cells in the CK-treated group versus the untreated control.

### 2.4. Cell Cycle Analysis

Based on Kim et al.’s paper [25], cells (1 × 10^6^ cells/mL) were treated with CK (0, 5 or 10 γM) for 24 h, incubated with RNase A (10 mg/mL) for 1 h at 37 °C and stained with propidium iodide (50 μg/mL) for 30 min. The DNA content of stained cells was analyzed by using FACSCalibur (Becton Dickinson, Franklin Lakes, NJ, USA) using CellQuest Software.

### 2.5. Western Blotting

Based on Koo et al.’s paper [26], cells (1x10^6^ cells/mL) were treated with various concentrations of CK for 24 h, followed by a general immunoblotting method. Isolated proteins were transferred to a Hybond ECL transfer membrane for detection with antibodies for PARP, Caspase-3, p-STAT3 (Tyr705), p-STAT3 (ser727), STAT3 (Cell signaling Technology, Beverly, MA, USA), PD-L1, c-Myc (Abcam, Cambridge, UK) and VEGF, TGF-β, Cyclin D1, Bcl-xL (Santa Cruz Biotechnology, Dallas, TX, USA) and β-actin (Sigma, St. Louis, MO, USA).

### 2.6. RT-qPCR Analysis

Based on Lee et al.’s paper [27], with the total RNAs isolated from CK-treated DU145 cells by using RNeasy mini kit (Qiagen), quantitative reverse transcription PCR (RT-qPCR) was carried out under the LightCycler TM instrument (Roche Applied Sciences, Indianapolis, IN, USA) by using the following primers, IL-6- forward: 5′-CCACCGGGAACGAAAGAGAA−3‘; reverse-: 5′-GAGAAGGCAACTGGACCGAA–3′ (Bioneer, Daejeon, Korea); IL-10 forward: 5′-TCTCCGAGATGCCTTCAGCAGA-3′; reverse-: 5′-TCAGACAAGGCTTGGCAACCCA−3′ (Bioneer, Daejeon, Korea); hGAPDH-forward: 50 -CCA CTC CTC CAC CTT TGA CA-30; reverse-: 50 -ACC CTG TTG CTG TAG CCA −3 0 (Bioneer, Daejeon, Korea).

### 2.7. Co-Immunoprecipitation

DU145 cells were lyzed in lysis buffer and then were immunoprecipitated with STAT3 antibody or normal immunoglobulin G antibody. Protein A/G sepharose beads (Santa Cruz Biotechnology, Santa Cruz, CA, USA) were also applied, and the precipitated proteins were subjected to immunoblotting with the antibodies of PD-L1 and STAT3.

### 2.8. Statistical Analysis

All values were expressed as means ± standard deviation (SD). For statistical analysis, Student’s *t*-test was used for the comparison of two groups by using GraphPad Prism software (Version 5.0, San Diego, CA, USA). The statistically significant difference was determined at *p*-values of < 0.05 between the control and CK-treated groups.

## 3. Results

### 3.1. Cytotoxic Effect of Compound K in Human Prostate Cancer Cells

To assess the cytotoxic effect of compound K (CK) (Figure 1a), cell viability assay was carried out in DU145, PC3 and LNCaP human prostate cancer cells exposed to indicated concentrations of CK (0, 2.5, 5 and 10 μM) by using MTT assay. Here CK inhibited the viability in DU145, PC3 and LNCaP cells (Figure 1b); DU145 cells were more susceptible to the cytotoxic effect of CK compared with PC3 and LNCaP cells. However, CK did not hurt the viability of RWPE1 normal prostate cells.

### 3.2. Correlation between STAT and PD-L1 in Prostate Cancer Cells

**Figure 2:**
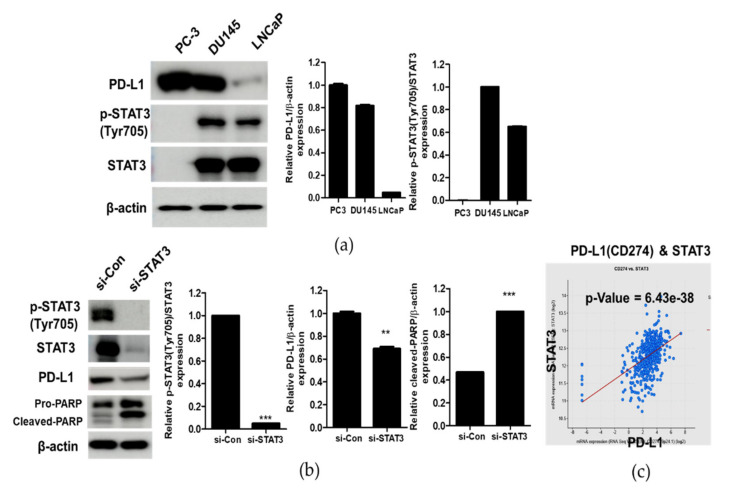
Correlation between STAT and PD-L1 in DU145, PC3 and LNCaP prostate cancer cells. (**a**) Endogenous expression levels of PD-L1 and p-STAT3 in PC3, DU145 and LNCaP prostate cancer cells. The expression levels of p-STAT3, STAT3 and PD-L1 in PC3, DU145 and LNCaP cells by Western blotting using anti-p-STAT3, STAT3 and PD-L1 antibodies. (**b**) Effect of STAT3 depletion on pro-PARP, cleaved-PARP and PD-L1 in DU145 cells. Cell lysates were prepared and subjected to Western blotting for p-STAT3, STAT3, PD-L1 and PARP. (**c**) cBioportal database shows the correlation between STAT3 and PD-L1. Graphs represent relative levels of protein/β-actin. Data represent means ± SD. ** *p* < 0.01 and *** *p* < 0.001 versus control.

To test the expression of PD-L1 and p-STAT3 (Tyr705) in DU145, PC3 and LNCaP prostate cancer cells, Western blotting was performed with the antibodies of PD-L1 and STAT3 (Tyr705). As shown in Figure 2a, PD-L1 was highly expressed in PC3 and DU145, while it was lowly expressed in LNCaP cell lines. Further, p-STAT3 was highly expressed in DU145 and LNCaP cells.

Considering that Du145 cells were more sensitive to the cytotoxic effect of CK compared to PC3 and LNCaP cells (Figure 1b), we hypothesize that the endogenous expression of p-STAT3 and PD-L1 in DU145 cells may be associated with better cytotoxicity in DU145 cells. Hence, to confirm whether STAT3 depletion is associated with PD-L1 and PARP, Western blotting was conducted in DU145 cells. As shown in Figure 2b, the depletion of STAT3 attenuated the expression of PD-L1 and increased the cleavage of PARP in DU145 cells. Furthermore, cBioportal correlation data demonstrate the close correlation between STAT3 and PD-L1 with spearman’s correlation coefficient, 0.53 (Figure 2c).

### 3.3. Compound K Effectively Attenuated the Expression of p-STAT3 and PD-L1 in DU145, PC3 and LNCaP Prostate Cancer Cells

**Figure 3:**
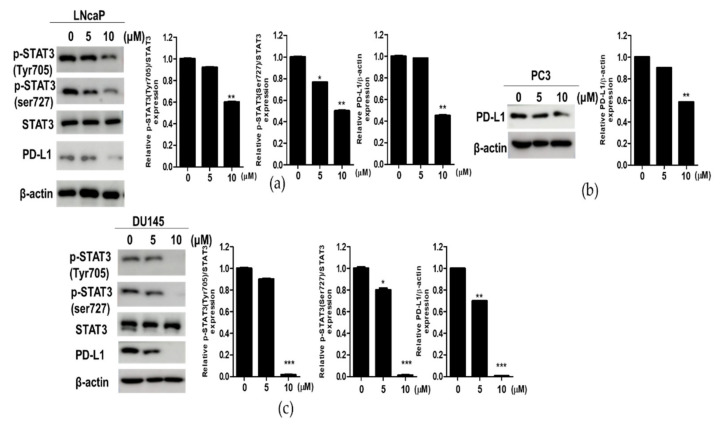
Effect of compound K on the expression of p-STAT3 (Tyr705), p-STAT3 (ser727), STAT3 and PD-L1 in prostate cancer cells. (**a**) LNCaP cells were treated with CK (0, 5 or 10 µM) for 24 h and subjected to Western blotting for p-STAT3 (Tyr705), p-STAT3 (ser727), STAT3 and PD-L1. (**b**) PC3 cells were treated with CK (0, 5 or 10 µM) for 24 h and subjected to Western blotting for PD-L1. (**c**) DU145 cells were treated with CK (0, 5 or 10 µM) for 24 h and subjected to Western blotting for p-STAT3 (Tyr705), p-STAT3 (ser727), STAT3, PD-L1 and β-actin. Graphs represent relative levels of protein/β-actin. Data represent means ± SD. * *p* < 0.05, ** *p* < 0.01 and *** *p* < 0.001 versus untreated control.

To confirm the effect of CK on STAT3 and PD-L1, Western-blotting was performed in DU145, PC3 and LNCaP cells treated with CK. As shown in Figure 3a,b, CK weakly attenuated the expression of p-STAT3 and PD-L1 in LNCaP and PC3 cells. In contrast, CK effectively suppressed the expression of p-STAT3 and PD-L1 in DU145 cells (Figure 3c).

### 3.4. Compound K Induced Apoptosis in DU145 Cells

**Figure 4:**
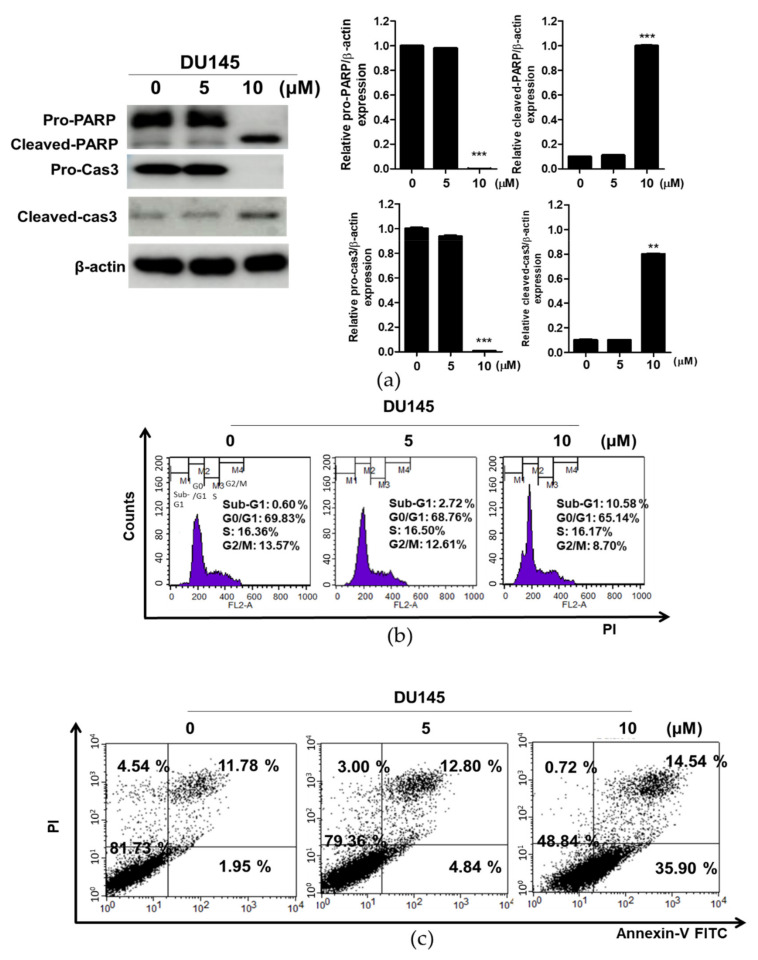
Effect of compound K on apoptosis in DU145 cells. (**a**) DU145 cells were treated with CK (0, 5 or 10 µM) for 24 h and subjected to Western blotting for pro-PARP, cleaved-PARP, pro-caspase-3 and cleaved caspase-3. (**b**) DU145 cells treated with CK (0, 5 or 10 µM) for 24 h were fixed with 70% ethanol, stained with propidium iodide (PI) for cell cycle distribution by flow cytometry. (**c**) DU145 cells were treated with CK (0, 5 or 10 μM) for 24 h. The cells were stained using FITC-Annexin V/PI dye staining for detecting early and late apoptotic portions by flow cytometry. Graphs represent relative levels of protein/β-actin. Data represent means ± SD. ** *p* < 0.01 and *** *p* < 0.001 versus untreated control.

To confirm the apoptotic effect of CK, Western blotting and cell cycle assay were performed in DU145 cells treated by CK. CK increased the cleavage of PARP and caspase-3, attenuated the expression of pro-PARP and pro-caspase-3 (Figure 4a) and also effectively increased the sub-G1 population in DU145 cells (Figure 4b). Consistently, a cell apoptosis assay using Annexin-V/PI double staining revealed that CK (10 μM) significantly increased the early and late apoptosis to 35.90% and 14.54% in DU145 cells, respectively, compared to the control (Figure 4c).

### 3.5. Compound K Effectively Attenuated the Expression of p-STAT3 and PD-L1 in INF-γ Stimulated DU145 Cells

**Figure 5:**
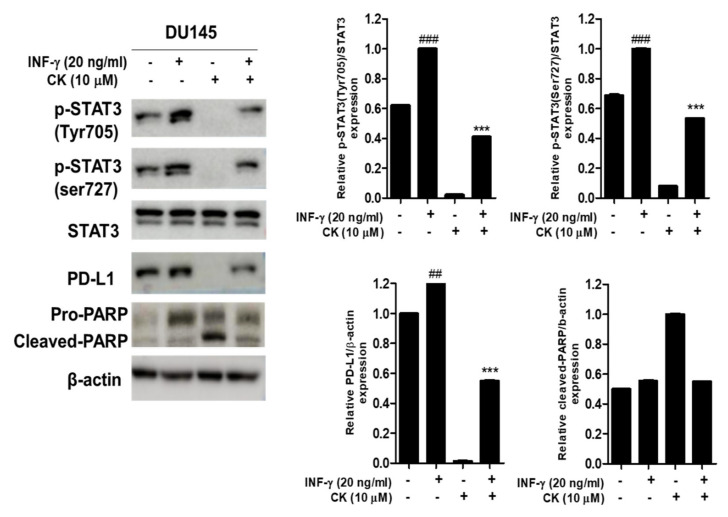
Effect of compound K on INF-γ stimulated p-STAT3 and PD-L1 in DU145 cells. DU145 cells were treated with 10 µM CK and 20 ng/mL recombinant human INF-γ protein for 24 h and were subjected to Western blotting for p-STAT3 (Tyr705), p-STAT3 (ser727), STAT3, PD-L1 and β-actin. Graphs represent relative levels of protein/β-actin. Data are shown as means ± SD. ## *p* < 0.01 vs. untreated control. ### *p* < 0.001 vs. untreated control. *** *p* < 0.001 versus INF-γ treated control.

IFN-γ is known to signal through the transcription factor STAT3 [28]. To evaluate the potential role of STAT3 activation in mediating PD-L1 protein expression, recombinant human INF-γ protein was used in CK-treated DU145 cells. Consistently, CK attenuated the expression of p-STAT3 (Tyr705), p-STAT3 (sre727), PD-L1 and pro-PARP at 10 µM in INF-γ stimulated DU145 cells (Figure 5).

### 3.6. Compound K Effectively Attenuated the Expression of Immune Escape-Related Proteins and STAT3 Target Proteins in DU145 Cells

**Figure 6:**
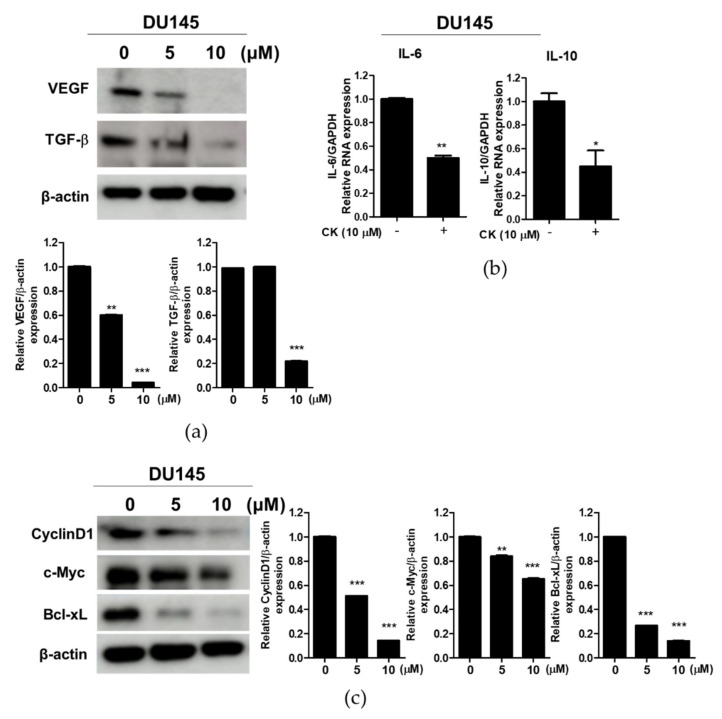
Effect of compound K on immune escape-related proteins and STAT3 target proteins in DU145 cells. (**a**) DU145 cells were treated with CK (0, 5 or 10 µM) for 24 h. Cell lysates were prepared and subjected to Western blotting for VEGF and TGF-β. (**b**) IL-6 and IL-10 mRNA expression in DU145 cells by qRT-PCR. (**c**) DU145 cells were treated with CK (0, 5 or 10 µM) for 24 h and subjected to Western blotting for CyclinD1, c-Myc and Bcl-xL. Graphs represent relative levels of protein/β-actin. Data stand for means ± SD. * *p* < 0.05, ** *p* < 0.01 and *** *p* < 0.001 versus untreated control.

To confirm whether or not the apoptotic effect of CK is related to STAT3 activation, Western blotting was conducted in CK-treated DU145 cells. As shown in Figure 6a, CK reduced the expression of VEGF, TGF-β, IL-6 and IL-10 as immune escape proteins in DU145 cells. Consistently, CK suppressed the expression of STAT3 target proteins, such as Cyclin D1, c-Myc and Bcl-xL, in DU145 cells (Figure 6b).

### 3.7. CK Disturbed the Interaction between STAT3 and PD-L1 in DU145 Cells

**Figure 7:**
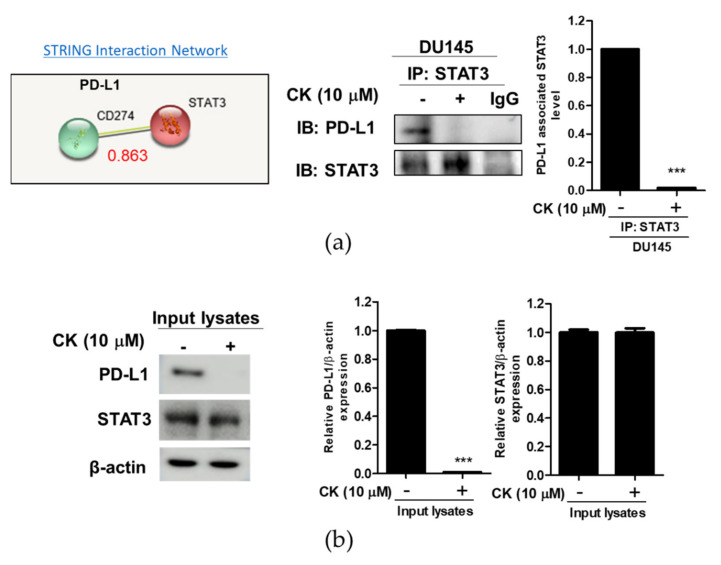
Effect of compound K on the interaction between STAT3 and PD-L1 in DU145 cells. (**a**) STAT3 interacts with PD-L1 by STRING database (interaction score: 0.863). Programmed death-ligand 1 (PD-L1) is also known as a cluster of differentiation 274 (CD274). (**b**) DU145 cells were treated with CK for 24 h and subjected to immunoprecipitation (IP) by using thelysates of DU145 cells with anti-STAT3 antibody or negative control IgG, and then Western blotting to detect PD-L1 in whole-cell lysates. Graphs show relative levels of protein/β-actin. Data stand for means ± SD. *** *p* < 0.001 versus untreated control.

To confirm the inhibitory effect of CK on the interaction between STAT3 and PD-L1, immunoprecipitation was conducted in the DU145 cell treated by CK. By using the String database, the binding score of protein–protein interaction between STAT3 and PD-L1 was known to be 0.863 (Figure 7a). As shown in Figure 7b, CK suppressed the binding of STAT3 with PD-L1 in DU145 cells.

### 3.8. miR193a-5p Plays a Critical Role in the CK-Induced Apoptosis in DU145 Cells

**Figure 8:**
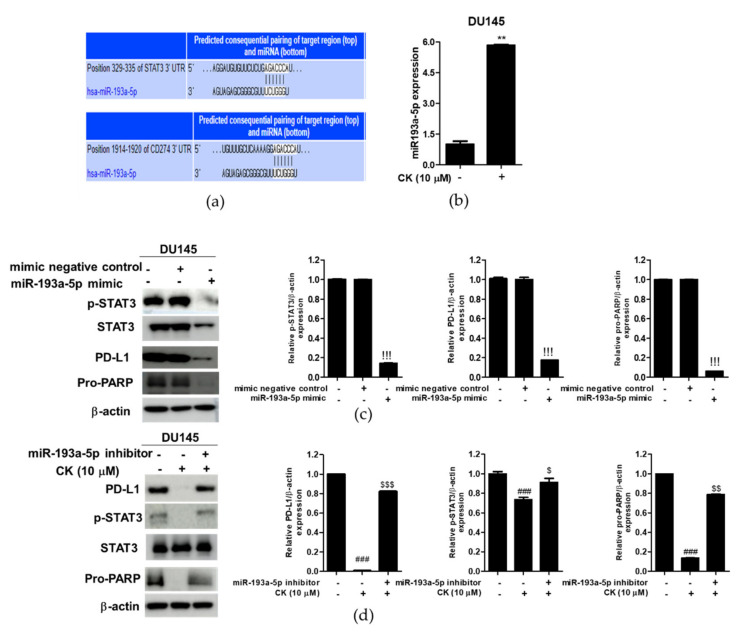
The important role of miR-193a-5p in CK-induced apoptosis in DU145 cells. (**a**) The TargetScan web server demonstrates that miR-193a-5p directly binds to the 3′-untranslated region of STAT3 and PD-L1. (**b**) Effect of CK on miR193a-5p expression by qRT-PCR in DU145 cells. (**c**) Effect of miR193a-5p mimic on p-STAT3, STAT3, PD-L1 and pro-PARP in DU145 and cells. (**d**) Effect of miR193a-5p inhibitor on p-STAT3, STAT3, PD-L1, pro-PARP in DU145 cells. Data are shown as means ± SD. ** *p* < 0.001 versus untreated control. !!! *p* < 0.001 versus mimic negative control. ### *p* < 0.001 vs. untreated control. ^$^ *p* < 0.05, ^$$^ *p* < 0.01 and ^$$$^ *p* < 0.001 versus CK-treated control.

To determine the role of miR193a-5p in CK-induced apoptosis, Western blotting was conducted in DU145 cells. TargetScan web server as a bioinformatic tool for predicting biological targets of microRNA demonstrates that miR-193a-5p directly binds to the 3′-untranslated region of STAT3 and PD-L1 (Figure 8a). As shown in Figure 8b, CK increased the mRNA expression of miR193a-5p in DU145 cells (Figure 8b). Furthermore, miR193a-5p mimic attenuated the expression of p-STAT3, STAT3, PD-L1 and pro-PARP in DU145 cells (Figure 8c). In contrast, miR193a-5p inhibitor reversed the ability of CK to suppress the expression of p-STAT3, PD-L1 and pro-PARP in DU145 cells (Figure 8d).

## 4. Discussion

In the present study, the apoptosis mechanism of CK, a metabolite of processed ginseng (Black ginseng) saponins, was explored in prostate cancer cells in correlation with STAT3 and PD-L1 signaling. Recent evidence demonstrates that STAT3 and PD-L1 are involved in a variety of biological processes, such as cancer growth, metastasis and modulation of the immune microenvironment in cancer cells [9,22].

It is well documented that STAT3 signaling plays an important role in tumor–immune cell interaction, cancer progression, metastasis, cell proliferation and immune escape [6,14,22,29]. Furthermore, STAT3-mediated cytokines are well-known to regulate components of the tumor microenvironment [14,30] and also mediate crosstalk between tumor cells and immune cells, including CD8+ T-cells, Tregs and NK cells [5,31].

Thus, the novel apoptotic mechanism of CK in association with STAT3 and PD-L1 signaling was examined in prostate cancer cells.

Interestingly, DU145 cells were more sensitive to the cytotoxic effect of CK compared to PC3 and LNCaP cells, while STAT3 and PD-L1 were overexpressed in DU145 cells more than in LNCaP and PC3 cells, implying the potent antitumor effect of CK in DU145 cells. Consistently, CK enhanced the cleavage of PARP, reduced the expression of pro-PARP, pro-caspase-3 and increased the sub G1 population in DU145 cells, indicating the cytotoxicity of CK is induced possibly via the apoptotic effect of CK in prostate cancer cells.

Accumulating evidence reveals that STAT3 directly activates transcription of VEGF, TGF-β, IL-6 and IL-10, leading to immune escape [29,32,33]. Herein, CK attenuated p-STAT3 (Tyr705) and p-STAT3 (ser727) in DU145 cells and also attenuated VEGF, TGF-β, IL-6 and IL-10 in DU145 cells, implying the apoptotic effect of CK via regulation of STAT3 and its related immune escape-related genes. Furthermore, CK suppressed STAT3 target genes, including CyclinD1 as a cell cycle protein, Bcl-xL as an anti-apoptotic protein, c-Myc as a proliferative protein [34,35] in DU145 cells, indicating the antitumor effect of CK via the inhibition of STAT3 target genes, including CyclinD1 and Bcl-xL.

PD-1 and its ligand PD-L1 are transmembrane proteins involved in autoimmunity, infection and antitumor immune response [36]. PD-L1 is overexpressed in tumor cells, while its receptor PD-1 is expressed in activated T-cells [37]. Hence, PD-L1/PD-1 combination leads to T-cell inactivation and the damage of effective immune response against tumor cells [38]. Emerging evidence reveals that PD-L1 overexpression is related to poor prognosis for cancer patient survival [39] and resistance to anti-cancer treatments [38,40]. Here, CK attenuated PD-L1 in DU145 cells, implying the inhibition of PD-L1 mediates CK induced apoptosis in DU145 cells.

Recent evidence reveals that STAT3 binds to the PD-L1 promoter and induces PD-L1 transcription activation to regulate tumor cells and the tumor-associated immune environment [9]. Moreover, STAT3 promotes immunosuppression by the upregulation of PD-L1, while overexpression of PD-L1 is significantly associated with the expression of phosphorylated STAT3 [9,41]. Likewise, phosphorylation of the STAT3 is correlated with the upregulation of PD-L1 in various cancers [6,12], and also cBioportal correlation data reveal the significant correlation between STAT3 and PD-L1 by spearman’s correlation coefficient (0.53). Consistently, the depletion of STAT3 attenuated the expression of PD-L1 and increased the cleavage of PARP in CK-treated DU145 cells, demonstrating the close interaction between STAT3 and PD-L1, which was suppressed by CK.

Accumulating evidence reveals that IFN-γ is a known mediator of PD-L1 expression through the transcription factor STAT3 in human cancer cells, leading to suppression of antitumor immune responses [10,42]. In the current study, CK reduced the expression of p-STAT3 (Tyr705), p-STAT3 (ser727), PD-L1 and pro-PARP in IFN-γ treated DU145 cells, demonstrating CK induced apoptosis via the inhibition of STAT3 and PD-L1 signaling.

Furthermore, to confirm the interaction between STAT3 and PD-L1, an immunoprecipitation assay was conducted in DU145 cells. Here, CK disturbed the binding of STAT3 and PD-L1 in CK induced apoptosis, confirming the apoptotic effect of CK by disturbing the binding of STAT3 and PD-L1.

It is well-known that many miRNAs are involved in tumor proliferation and suppression [43]. Recent evidence reveals that miR193a-5p induces apoptosis and inhibits proliferation in DU145 and PC3 prostate cancer cells [44]. Consistently, CK upregulated the mRNA expression of miR193a-5p in DU145 cells, and miR193a-5p mimic suppressed the expression of p-STAT3, STAT3, PD-L1 and pro-PARP, whereas miR193a-5p inhibitor reversed the ability of CK to attenuate p-STAT3, PD-L1 and pro-PARP in DU145 cells, demonstrating the critical role of miR193a-5p in CK induced apoptosis in DU145 cells.

## 5. Conclusions

**Figure 9:**
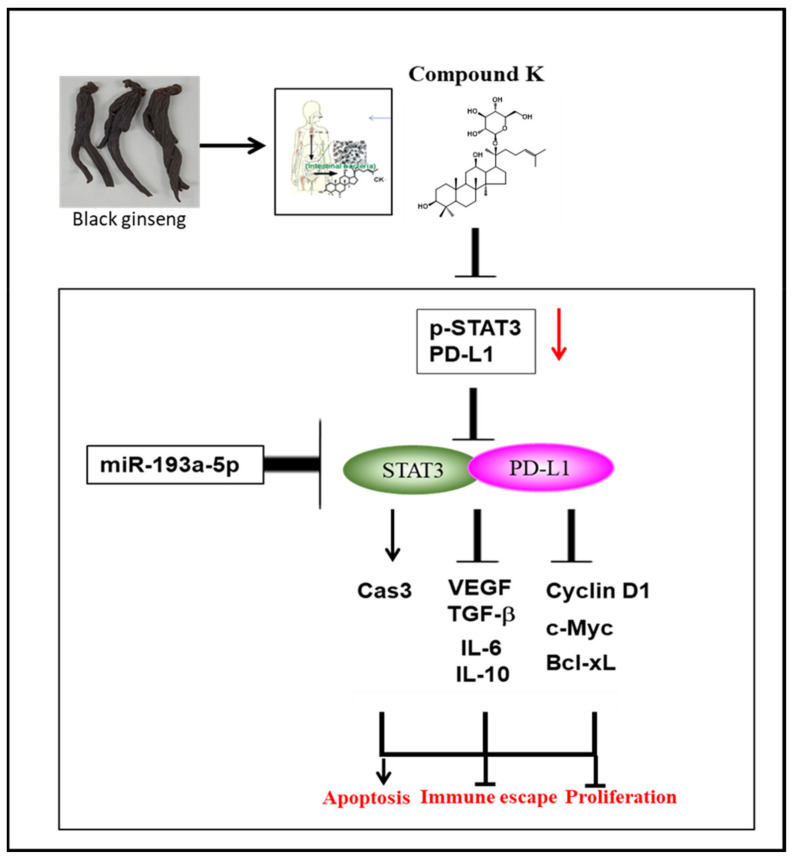
Schematic diagram of the apoptotic effect of compound K via the activation of miR193a-5p and inhibition of STAT3/PD-L1 signaling.

In summary, CK increased cytotoxicity without hurting normal cells, sub G1 population, cleaved PARP and decreased the expression of pro-caspase-3, VEGF, TGF-β, IL-6, IL-10, Cyclin D1, c-Myc and Bcl-xL in prostate cancer cells. Furthermore, CK suppressed the expression of p-STAT3 and PD-L1 and disturbed the binding of STAT3 and PD-L1 in DU145 cells. CK attenuated the expression of INF-γ stimulated p-STAT3, PD-L1 and pro-PARP in DU145 cells. Furthermore, CK upregulated miR193a-5p, and the miR193a-5p inhibitor reversed the ability of CK to attenuate the expression of p-STAT3, PD-L1 and pro-PARP in DU145 cells. Taken together, our findings provide a highlight that the activation of miR193a-5p and the inhibition of PD-L1 and STAT3 are critically involved in the apoptotic effects of CK in DU145 cells (Figure 9).
